# Expression of MATE1, P-gp, OCTN1 and OCTN2, in epithelial and immune cells in the lung of COPD and healthy individuals

**DOI:** 10.1186/s12931-018-0760-9

**Published:** 2018-04-20

**Authors:** Tove Berg, Tove Hegelund-Myrbäck, Johan Öckinger, Xiao-Hong Zhou, Marie Brännström, Michael Hagemann-Jensen, Viktoria Werkström, Janeric Seidegård, Johan Grunewald, Magnus Nord, Lena Gustavsson

**Affiliations:** 1 Respiratory Medicine Unit, Department of Medicine Solna and Center for Molecular Medicine, Karolinska Institutet, Stockholm, Sweden; 2 Quantitative Clinical Pharmacology, Early Clinical Development, IMED Biotech Unit, AstraZeneca R&D, Gothenburg, Sweden; 3 Respiratory, Inflammation and Autoimmunity, IMED Biotech Unit, AstraZeneca R&D, Gothenburg, Sweden; 4 Respiratory GMed, Global Medicines Development, AstraZeneca R&D, Gothenburg, Sweden; 5 Global Patient Safety, Global Medicines Development, AstraZeneca R&D, Gothenburg, Sweden; 6 Department of Drug Metabolism, H. Lundbeck A/S, Ottiliavej 9, 2500 Valby, Denmark

**Keywords:** MATE1, P-gp, OCTN1, OCTN2, Immunohistochemistry, COPD, Human lung tissue, Drug transporters, Uptake, Efflux

## Abstract

**Background:**

Several inhaled drugs are dependent on organic cation transporters to cross cell membranes. To further evaluate their potential to impact on inhaled drug disposition, the localization of MATE1, P-gp, OCTN1 and OCTN2 were investigated in human lung.

**Methods:**

Transporter proteins were analysed by immunohistochemistry in lung tissue from healthy subjects and COPD patients. Transporter mRNA was analysed by qPCR in lung tissue and in bronchoalveolar lavage (BAL) cells from smokers and non-smokers.

**Results:**

We demonstrate for the first time MATE1 protein expression in the lung with localization to the apical side of bronchial and bronchiolar epithelial cells. Interestingly, MATE1 was strongly expressed in alveolar macrophages as demonstrated both in lung tissue and in BAL cells, and in inflammatory cells including CD3 positive T cells. P-gp, OCTN1 and OCTN2 were also expressed in the alveolar epithelial cells and in inflammatory cells including alveolar macrophages. In BAL cells from smokers, MATE1 and P-gp mRNA expression was significantly lower compared to cells from non-smokers whereas no difference was observed between COPD patients and healthy subjects. THP-1 cells were evaluated as a model for alveolar macrophages but did not reflect the transporter expression observed in BAL cells.

**Conclusions:**

We conclude that MATE1, P-gp, OCTN1 and OCTN2 are expressed in pulmonary lung epithelium, in alveolar macrophages and in other inflammatory cells. This is important to consider in the development of drugs treating pulmonary disease as the transporters may impact drug disposition in the lung and consequently affect pharmacological efficacy and toxicity.

**Electronic supplementary material:**

The online version of this article (10.1186/s12931-018-0760-9) contains supplementary material, which is available to authorized users.

## Background

Inhaled administration is the main treatment route in chronic obstructive pulmonary disease (COPD), enabling high local drug concentrations, lower systemic exposure and consequently, less risk of side effects. The inhalation device configuration and drug particle size affect the drug deposition pattern in the lung [1, 2]. The mechanisms that govern pulmonary drug disposition after inhalation, such as dissolution, uptake and elimination are however less well understood and are also affected by disease pathology. Knowledge on the parameters affecting drug disposition in the lung is important when predicting tissue retention and the likelihood of reaching sufficient drug concentrations at the pharmacological target.

Membrane transporters have proven important in drug disposition, and consequently in drug efficacy and toxicity [3]. However, drug transporter expression and function in the lung is still an emerging area [4–6]. Several drug transporter genes are known to be expressed in the lung [7, 8], for example organic cation transporters (e.g. OCT1, OCT3, OCTN1, OCTN2), and organic anion transporting polypeptides (e.g. OATP2A1, OATP2B1, OATP3A1, OATP4A1, OATP4C1) [7, 8]. Similarly, mRNA expression of transporters of the ATP-binding cassette family has been described in lung, including the multidrug resistance proteins (e.g. P-glycoprotein (P-gp)), breast cancer resistance protein (BCRP) and multidrug-resistance associated proteins (e.g. MRP1) [7, 8]. The expression of some of the transporters above was also confirmed on protein level through quantification by mass spectrometry [9].

In the present paper, we have focused on the expression of organic cation transporters OCT1, OCTN1, OCTN2 and MATE1, as well as P-gp. OCT1, OCTN1 and OCTN2 are mainly involved in cellular uptake of compounds, whereas MATE1 secretes organic cations out of cells. P-gp excretes drugs out of cells by an ATP-dependent mechanism. In particular the organic cation transporters, OCT1, OCTN1 and OCTN2 have been found to transport inhaled drugs e.g. muscarinic antagonists and β-adrenergic agonists [10–13], several of which have poor passive permeability and therefore are dependent on transporters to cross cell membranes [6, 10]. It has been proposed that organic cation transporters are important for these drugs to cross the airway epithelial barrier to reach the smooth muscle cells [11, 14]. Furthermore, organic cation transporters have been demonstrated to impact the disposal of inhaled drugs. As an example, OCT3 facilitates uptake of β-adrenergic agonists into airway smooth muscle cells [15]. MATE1 substrate specificity is similar to OCT1 and OCT2 [16] and we have recently shown that this transporter is expressed in lung [7]. Notably, the muscarinic antagonist ipratropium was recently reported to be a substrate of MATE1 [17]. P-gp is also of interest since it has a broad substrate specificity including basic compounds, a common feature of inhaled drugs. Moreover, P-gp is known to transport some glucocorticosteroids [18, 19], commonly used in the treatment of COPD.

Due to the heterogenic structure of the lung, with highly specific expression patterns [6, 20], it is important to study the spatial distribution and cellular localization of pulmonary drug transporters. High, cell specific expression of a gene/protein, could be masked by low average whole organ expression. In immunohistochemistry (IHC) studies, P-gp has been detected in the apical membrane of bronchial and bronchiolar epithelium and to some degree in alveolar macrophages [21]. Furthermore, OCTN1 and OCTN2 were expressed in respiratory epithelial cells [11], OCTN1 predominantly in the central airways whereas OCTN2 was focused peripherally, *e.g.*in alveolar epithelia [11], where these transporters may contribute to absorption of cationic bronchodilators [11–13]. We have recently investigated the mRNA expression of several membrane transporters in pulmonary tissue and detected similar expression levels in the lungs from ex-smoker COPD patients compared to healthy lung tissue [7]. However, little is known about the cell specific expression pattern of membrane transporters, e.g. in inflammatory cells.

COPD is associated with chronic inflammation, tissue destruction, progressive loss of lung function and airway obstruction. Smoking is the main cause of COPD, estimated to be the third leading cause of death in 2020 [22, 23]. Alveolar macrophages are key players in the inflammatory response and their quantity is increased in the lungs of COPD patients and healthy smokers, compared to non-smokers [24–27]. Alveolar macrophages secrete both pro- and anti-inflammatory cytokines, and components promoting emphysema, fibrosis, and mucohypersecretion [28–30]. Hence, alveolar macrophages, and other inflammatory cells, are interesting targets for drug development. The expression of drug transporters on the surface of these cells could affect drug concentration at the site of the target. A better understanding of the transporter expression in healthy and diseased lung would facilitate the development of successful drugs for COPD and other lung diseases.

This study aimed at investigating the pulmonary cellular expression patterns of the human membrane transporters OCT1, OCTN1, OCTN2, MATE1 and P-gp. IHC analyses with specific anti-bodies were performed on lung biopsies from ex-smokers with severe stage of COPD and healthy non-smokers. Furthermore, drug transporter expression in bronchoalveolar lavage as well as in a model of macrophages was investigated.

## Methods

### Human tissue material and IHC

The human material included for IHC studies was fully described previously [7, 31, 32] and demographic details are summarized in Additional file 1: Table S1. Briefly, the biopsy material from three healthy subjects and seven patients with severe COPD who all underwent lung transplantation were included. Healthy controls were in matched age intervals. All COPD subjects were ex-smokers and healthy controls were non-smokers. Biopsies were obtained from the bronchi and peripheral tissue, and the tissue samples were fixed in formalin, immediately after sampling. The study was approved by the Regional Ethical Review Board (Lund, Sweden) and all subjects gave written and oral informed consent.

For IHC the formalin fixed tissues were embedded in paraffin, and cut in 4-μm sections. Antigen retrieval was performed in the microwave for 30 min with 10 mM citrate buffer pH 6 (α-MATE1 and α-P-gp) or Tris-EDTA pH 9.0 (10 mM Tris Base, 1 mM EDTA Solution, α-OCTN1 and α-OCTN2). The endogenous biotin activity was blocked by using the Avidin and Biotin Blocking kit (Vector Laboratories, Burlingame, CA). The endogenous peroxidase activity was blocked in 0.3% H_2_O_2_ in phosphate buffered saline (PBS) for 30 min at room temperature. The slides were then blocked in PBS with 0.2% Tween, 5% serum, and 2% bovine serum albumin and incubated with the primary antibodies over night at 4 °C. After washing, the slides were incubated with the biotinylated secondary antibody for 2 h at room temperature. Immune complexes were detected with Vectastain ABC Kit (Vector Laboratories, Burlingame, CA) and 3,3′-diaminobenzidine (DAB) tablets (Sigma, St. Louis, MO). Nuclei were briefly counterstained with Mayer’s hematoxylin (Histolab, Stockholm, Sweden). For all stainings an IgG control were used using the equivalent antibody concentration. Pictures were taken with Nikon Eclipes Ni microscope with a Nikon DS-Ri2 camera (Nikon, Dusseldorf, Germany) and images were compared using Adobe Photoshop (Adobe, San Jose, CA).

For co-staining studies, the slides were treated as described above followed by incubation with the first primary antibodies, P-gp, MATE1, OCTN1 and OCTN2, respectively, and then the biotinylated secondary antibodies. Then the slides were incubated with StreptAvidin Alexa 488 for 45 min at room temperature. After washing in PBS, the procedure was repeated to block endogenous biotin activity and followed by the incubation with CD3 (T cell marker), CD20 (B cell marker), p63 (basal cell marker) and Thyroid Transcription Factor1 (TTF1, alveolar type II cell marker) antibodies at 4 °C overnight. Goat anti mouse IgG Alexa Fluor 568 was incubated for 45 min at room temperature followed by 3 times washing in PBS. Slides were mounted in VectaSheild mounting medium with DAPI. The matched isotype IgG antibodies were used as the negative control (see figures in Additional file 1).

### Antibodies used for IHC

Primary antibodies used were P-gp (HPA002199, 1:200), Atlas Antibodies, Stockholm, Sweden), MATE1 (HPA021987, 1:200, Atlas Antibodies), OCTN1 (sc-19,819, 1:400, Santa Cruz, CA), OCTN2 (sc-19,822, 1:400, Santa Cruz), CD3 (M7254, Agilent, 1:50), CD20 (M0755, Agilent, 1:400), p63 (M7317, Agilent, 1:50), TTF1 (M3535, Agilent, 1:50). Biotinylated secondary antibodies were all from Vector Laboratories (Burlingame, CA) and the IgG antibodies used as controls were IgG rabbit (15006) and IgG goat (19149) both from Sigma, St. Louis, MO. Mouse IgG (I-2000) and VectaSheild mounting medium with DAPI (H-1200) were from Vector Laboratories. StreptAvidin Alexa 488 (S-11223, 1:500) and goat anti mouse IgG Alexa Fluor 568 (A-11004, 1:500) were both from Thermo Fisher Scientific (Waltham, MA, USA).

### Bronchoscopy and bronchoalveolar lavage

Bronchoalveolar lavage (BAL) was collected from 22 subjects (8 non-smokers and 14 smokers) (for further information, see Table 1) as previously described [33]. The study was approved by the Regional Ethical Review Board (Stockholm, Sweden) and all subjects gave written and oral informed consent. For inclusion in this part of the study the subjects should either smoke daily (≥ 5 cigarettes/day) i.e. *smokers,* alternatively not have smoked at all during the previous 12 months, and in total < 100 cigarettes in their lifetime i.e. *non*-*smokers*. All the subjects showed dynamic spirometry values within the normal range [34], as measured by Medikro PRO (Aiolos Medical, Karlstad, Sweden). None of the subjects had clinically relevant airway infections or allergy symptoms at the time of bronchoscopy. Subjects diagnosed with asthma, COPD or other lung diseases, or with other inflammatory conditions were not included in the study.

**Table 1:** Information on smokers and non-smokers with normal lung function, included in the study

	Non-Smokers	Smokers	*p*-value ^*f*^
Total (n)	8	14	
Males/Females (n)	3/5	3/11	
Age	25 (20–34)	25 (19–50)	ns
Smoke status			
Pack Years ^a^	na	6 (1.8–24.5)	
Cigarettes/day ^b^	na	10 (7–30)	
Spirometry			
FVC ^c^	102 (86–120)	108 (85–127)	ns
FEV ^d^	101 (89–113)	104 (80–121)	ns
FEV1/FVC	83 (81–88)	78 (76–88)	ns
BAL			
Recovery %	74 (64–80)	69 (50–77)	ns
Total cell count (10^6)	18.1 (10.1–24.4)	39.8 (15.9–154)	***
Cell conc. (10^6 cells /L)	94.0 (68.0–154.5)	257.8 (90.3–987.2)	**
Macrophages %	87.4 (66.6–96.0)	96.5 (92.0–99.0)	***
Macrophages (10^6 cells /L)	79.2 (60.5–101.6)	250.5 (83.1–949.7)	***
Lymphocytes %	10.5 (3.4–31.6)	1.9 (0.40–7.20)	***
Lymphocytes (10^6 cells /L)	8.75 (3.58–48.2)	6.40 (1.03–29.62)	ns
Neutrophils %	1.30 (0.20–6.40)	0.60 (0.20–2.40)	ns
Neutrophils (10^6 cells /L)	1.06 (0.14–5.98)	1.49 (0.34–8.95)	ns
Mastcells ^e^	2.00 (0.00–10.0)	1.00 (0.00–6.00)	ns

Numbers indicate median and range, if not otherwise indicated. Eosinophils and basophils were also counted and constituted ≤1.4% and ≤ 0.2%, respectively, of BAL cells in any subject, with no difference between smokers and non-smokers. ^a^ Pack years calculated as ([cigarettes smoked per day]/20 x [years smoking]). ^b^ Current smoking habits at time of bronchoscopy. ^c^ FVC: forced vital capacity, % of predicted. ^d^ FEV1: Forced expiratory volume in 1 s, % of predicted, ^e^ Calculated in 10 fields of view at 16× magnification. ^*f*^ Statistics calculated using Mann Whitney non-parametric test: ** < 0.01, *** < 0.001, ns: Not significant, na: Not applicable

BAL fluid Cells were pelleted by centrifugation at 400×g, 4 °C, for 10 min and the supernatants were removed. The cell pellets were resuspended in RPMI-1640 and counted. 1 × 10^6^ BAL cells were pelleted and stored at − 80 °C for isolation of RNA. Smears for differential counts were prepared by cytocentrifugation at 50×g for 3 min (Cytospin 2 Shandon; Southern Products Ltd.), followed by May-Grünwald-Giems staining.

### mRNA expression analyses

mRNA from lung tissues was extracted from ~ 3 × 3 × 7 mm specimens. The tissue was homogenized in a Tissue Lyser II (Qiagen GmbH, Hilden, Germany) with one pre-chilled steel ball for 30 s at 2000 rpm. Thereafter, 1 mL TRIZOL reagent (Life Technologies, Carlsbad, CA) was added to the pulverized tissue and the RNA was extracted according to the TRIZOL protocol provided by the manufacturer. mRNA from BAL cells was isolated using the Allprep DNA/RNA/Protein Mini kit (Qiagen) while mRNA from cultured, PMA differentiated THP-1 cells (for details see Additional file 1) was extracted using the RNeasy Plus Mini Kit (Qiagen). In all experiments, RNA was reverse transcribed using the High Capacity RNA-to-cDNA Kit (Applied Biosystems), and gene expression was analyzed in duplicates using real-time quantitative PCR (qPCR) on the ABI Prims 7700 or CFX384 Real Time System (Bio-Rad, CA). TaqMan® Gene Expression Assays (Life technologies, NY) were used for analyzing expression of genes encoding membrane transporters *ABCB1*: *Hs00184500_m1*, *SLC47A1*: *Hs00217320_m1*, *SLC22A1*: *Hs00427552_m1*, *SLC22A4*: *Hs01548718_m1*, and *SLC22A5*: *Hs00929869_m1*, as well as the house keeping gene, hypoxanthine phosphoribosyltransferase 1*, HPRT1*: *Hs02800695_m1*, with cycling conditions recommended by the manufacturer (95 °C for 10 min and 40–45 cycles of (95 °C for 15 s and 60 °C for 1 min), using Taqman gene expression Mastermix (Life technologies, NY). *HPRT-1* was used as endogenous control, and expression levels of investigated genes were calculated by the comparative Ct method (2^ΔΔCt), compared to healthy individuals (BAL cells) or unstimulated cells (THP-1 cells).

### Uptake studies in THP-1 cells

THP-1 cells were cultured as described in Additional file 1 and seeded in 24 well plates for uptake studies. The cells were differentiated with 10 ng/ml PMA to adherent macrophages overnight (for further details see Additional file 1). Experiments were conducted 48 or 72 h post PMA-removal. Donor solutions were prepared in HBSS with 25 mM HEPES, pH 7.4. [^14^C]-metformin (Moravek, Brea, CA, USA; final concentration 11 μM and 1 μCi/mL) and [^3^H]-digoxin (PerkinElmer, Waltham, MA, USA; final concentration 0.5 μM and 1 μCi/mL) were used as substrates and pyrimethamine (1 μM) and elacridar (10 μM) were used as the inhibitors for MATE-1 and P-gp, respectively. Solvent concentrations were kept identical with and without inhibitor, at a maximum concentration of 0.2%. Uptake studies were performed in a thermostatic shaker (THERMO star, Lab Technologies GmbH), set to 37°C and 250 rpm. Plates and solutions were pre-warmed, the cells washed with HBSS (Invitrogen) and pre-incubated for approximately 10 min with and without inhibitor. To the start the uptake, donor solution, containing substrate alone or substrate and inhibitor, was added to each well.

The uptake was stopped at designated time points, by immediate washing with ice-cold PBS, before lysing the cells with 0.2 M NaOH. An equimolar amount of HCl was added to each well in order to neutralize the samples before scintillation counting and protein determination. The radioactivity in cell lysates was analyzed using a liquid scintillation counter (Tri-Carb, Perkin Elmer, Waltham, MA, USA) after addition of HiSafe2 scintillation cocktail (PerkinElmer, Waltham, MA, USA) to the samples.

Total protein concentration in cell lysates was determined using the Pierce® BCA Protein Assay kit (Thermo Scientific, Waltham, MA, USA). The protein concentration was determined spectrophotometrically in a plate reader (SpectraMAX, Molecular Devices, Sunnyvale, CA, USA) at 562 nm.

Unpaired two-tailed t-tests comparing uptake of substrate alone and substrate with inhibitor present, were calculated for each time point using GraphPad Prism (version 6.01, GraphPad Software Inc., San Diego, CA, USA). *p*-values < 0.05 were considered to be significant.

## Results

### Localized differences in expression of membrane transporter mRNA

**Fig. 1:**
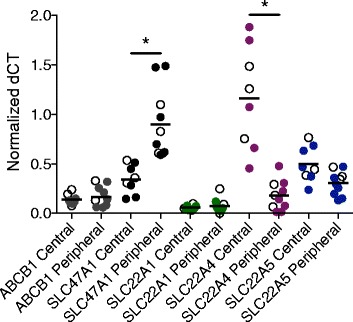
Comparison of drug transporter mRNA expression in central airways and peripheral lung tissue. Relative mRNA expression of *ABCB1*, *SLC47A1*, *SLC22A1*, *SLC22A4*, and *SLC22A5*, expressed as normalized dCT, in central and peripheral lung tissue from healthy subjects and ex-smokers with COPD. Filled circles denote ex-smokers with COPD, open circles denote healthy subjects, and horizontal lines indicate mean. Statistical significance of difference; * *p* < 0.05

We have previously found expression of the genes *ABCB1* encoding P-gp, *SLC47A1* encoding MATE1, *SLC22A1* encoding OCT1, *SLC22A4* encoding OCTN1, and *SLC22A5* encoding OCTN2 in both the central and peripheral regions of the lung of ex-smokers with severe stage of COPD and healthy controls [7]. No differences in mRNA expression for these transporters were observed between COPD patients and controls in this study. Among these genes *SLC47A1* (MATE1) displayed the strongest mRNA expression in the peripheral lung tissue [7]. We show that *SLC47A1* (MATE1) expression was significantly higher in peripheral lung tissue as compared to bronchi (Fig. 1). In contrast, *SLC22A4* (OCTN1) showed the strongest expression in bronchi and was expressed to a significantly lower degree in peripheral lung tissue (Fig. 1), while no difference was seen in mRNA expression levels of *ABCB1* (P-gp)*, SLC22A1* (OCT1) and *SLC22A5* (OCTN2) between the bronchi and peripheral lung (Fig. 1).

### Protein expression and cellular localization of membrane transporters in the lung

We further investigated the protein expression pattern and cellular localization of these transporters by IHC in biopsy material taken from locations adjacent to the biopsies extracted for RNA analyses. The main findings are outlined here below and summarized in Table 2 whereas further findings are presented in Additional file 1.

**Table 2:** Transporter protein expressions in human lung in COPD patients and healthy volunteers detected by immunohistochemistry

Tissue	P-gp		MATE1		OCTN1		OCTN2	
	HV	COPD	HV	COPD	HV	COPD	HV	COPD
Bronchial epithelial cells	x	x	x	x	x	x	x	x
Bronchiolar epithelial cells	x	x	x	x	x	x	x	x
Alveolar epithelial cells (type II cells)	x	x	nd	nd	x	x	x	x
Alveolar macrophages	x	x	x	x	x	x	x	x
Lymphocytes in bronchi^a^	nd	nd	x	x	nd	nd	nd	nd

^a^Infiltrating in epithelial layer, HV: Tissue from healthy volunteers, COPD: Tissue from COPD patients, X: Expression detected, nd: Not detected

**Fig. 2:**
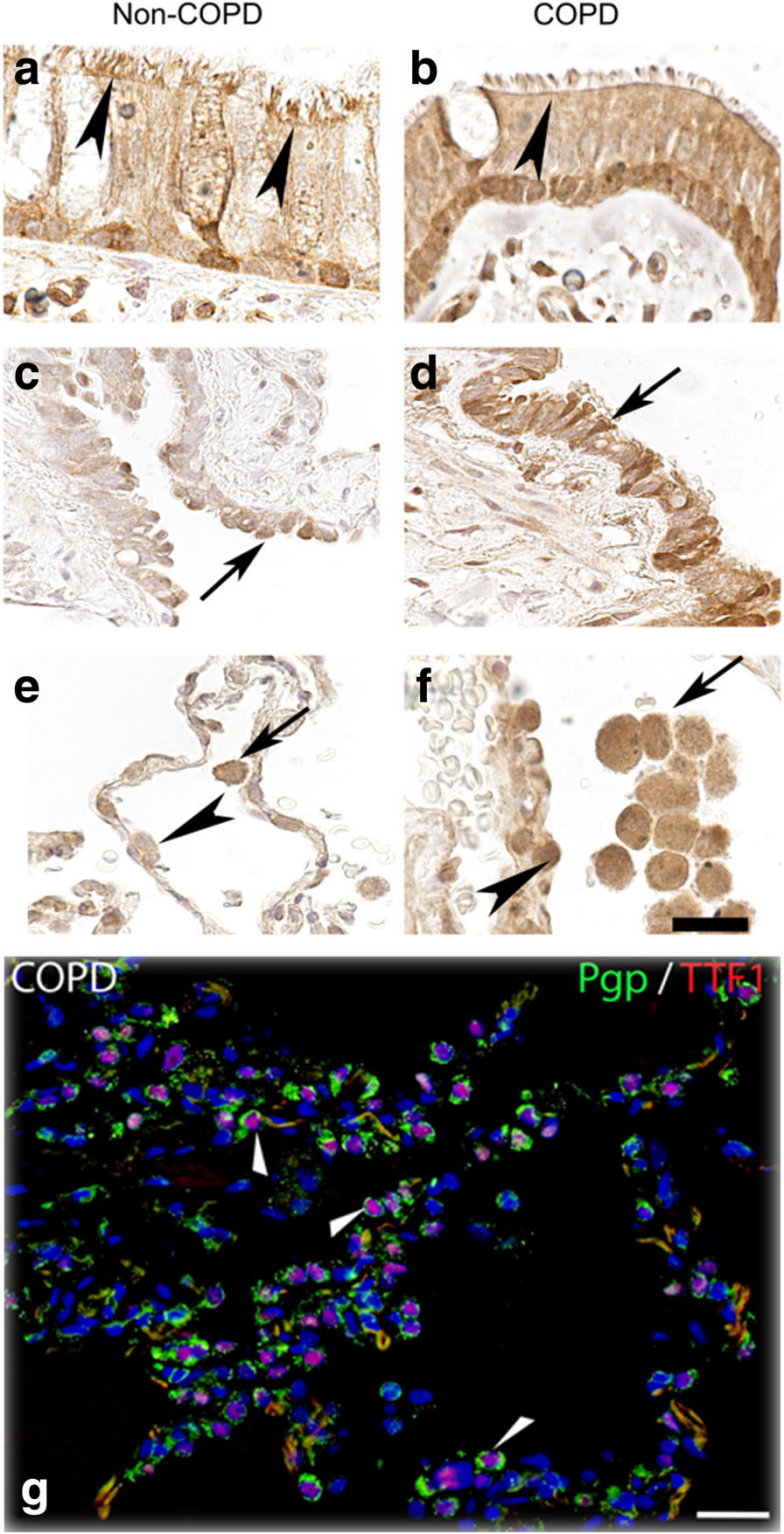
P-gp protein expression in airway and alveolar epithelial cells and in alveolar macrophages. Paraffin sections of human lung tissue from healthy individuals (left column) and ex-smokers with COPD (right column) were immunostained with anti- P-gp antibody (brown) and counterstained with hematoxylin (blue). **a** and **b** show P-gp positive signals in epithelial cells in bronchi (arrow heads). **c** and **d** show P-gp staining in the bronchiolar epithelium (arrows). **e** and **f** show P-gp expression in peripheral tissue where arrows indicate alveolar macrophages and arrow heads denotes alveolar epithelial cells. **g** show P-gp expression in alveolar type II cells (TTF1 positive) after co-staining with P-gp (green) and TTF1 (red) antibodies (arrow heads). Scale bar: 20 μm

P-gp protein was expressed in epithelial cells in bronchial airways with more profound staining on the apical cell membrane of ciliated cells (Fig. 2a and b), as well as in the bronchiolar epithelium (Fig. 2c and b). In the peripheral lung tissue, P-gp expression was found in alveolar macrophages as well as in alveolar epithelial cells (Fig. 2e and f), which were positive for the alveolar type II cells marker TTF1 (Fig. 2g). P-gp was also expressed in seromucous glands (Additional file 1: Figure S1A and B), endothelial cells (Additional file 1: Figure S1C and D) and nerve ganglia (data not shown).

**Fig. 3:**
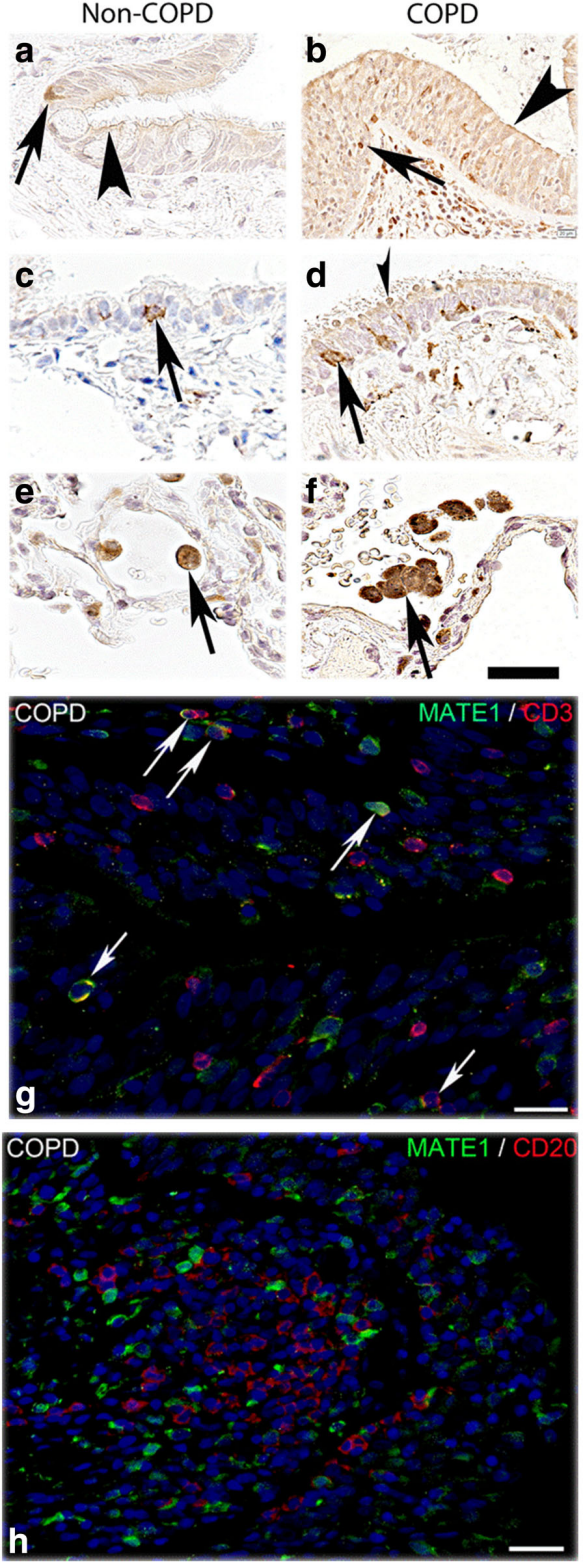
MATE1 protein expression in airway epithelial cells, alveolar macrophages and infiltrating T-cells. Paraffin sections of human lung tissue from healthy individuals (left column) and ex-smokers with COPD (right column) were immunostained with anti-MATE1 antibody (brown) and counterstained with hematoxylin (blue). **a** and **b** Show apical staining in bronchial epithelial cells and in **c** and **d** bronchiolar epithelium (arrow heads) as well as staining in some inflammatory cells migrating through the bronchial and bronchiolar epithelium (arrows). **e** and **f** Show strong expression of MATE1 in alveolar macrophages in the peripheral tissue (arrows). **g** and **h** Show co-staining of MATE1 antibody with T cell (CD3) and B cell (CD20) marker antibodies demonstrating that the infiltrating T cells (arrows) but not B cells are MATE1 positive. Scale bar: 20 μm

When analyzing the SLC transporters, we detected MATE1 expression on the apical membrane of epithelial cells of the bronchial and bronchiolar epithelium of both healthy subjects and COPD patients (Fig. 3a-d). A strong expression of MATE1 was observed in lymphocytes infiltrating to the epithelial layer in the bronchi (Fig. 3a-d). Immunofluorescent co-staining indicated that the infiltrating, MATE1 expressing, immune cells included T cells (CD3 positive), (Fig. 3g) but not B cells (CD20 positive) (Fig. 3h). MATE1 was also strongly expressed in alveolar macrophages in the peripheral lung (Fig. 3e and f). MATE1 expression was also noted in endothelial cells (Additional file 1: Figure S2A and B), as well as in smooth muscle cells and nerve ganglia (data not shown).

**Fig. 4:**
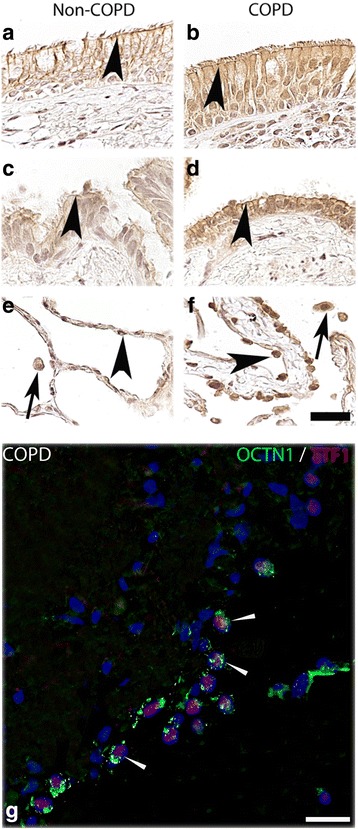
OCTN1 protein expression in airway and alveolar epithelial cells and in alveolar macrophages. Paraffin sections of human lung tissue from healthy individuals (left column) and ex-smokers with COPD (right column) were immunostained with anti-OCTN1 antibody (brown) and counterstained with hematoxylin (blue). **a** and **b** Show the OCTN1 expression on the apical side of the bronchial epithelium (arrow heads). **c** and **d** Show expression of OCTN1 in the bronchiolar epithelial cells (arrow heads). **e** and **f** The expression of OCTN1 in alveolar epithelial cells (arrow heads) and alveolar macrophages (arrows). **g** Shows the expression of OCTN1 in alveolar type II cells (TTF1 positive cells) after co-staining with OCTN1 (green) and TTF1 (red) antibodies. Scale bar: 20 µm

OCTN1 expression was found in the apical and lateral side (Fig. 4a and b) of the epithelial cells in the bronchial airways in both COPD and healthy subjects. OCTN1 was also expressed in epithelial cells lining the bronchioles (Fig. 4c and d) as well as in alveolar macrophages, and in epithelial cells in the peripheral lung (Fig. 4e and f). OCTN1 showed co-localization with TTF1 indicating expression in alveolar type II cells (Fig. 4g). OCTN1 was also expressed in seromucous glands (Additional file 1: Figure S3A and B) and endothelial cells (Additional file 1: Figure S3C and D).

**Fig. 5:**
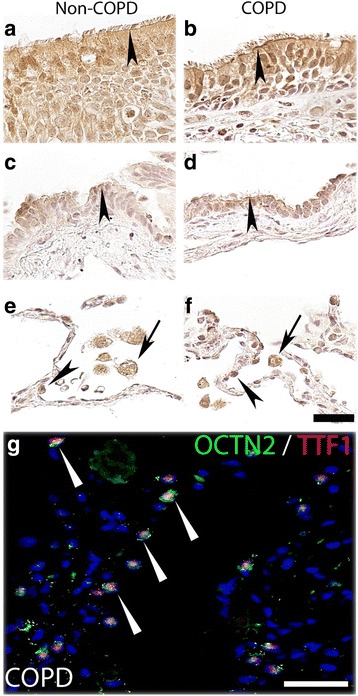
OCTN2 protein expression in airway and alveolar epithelial cells and in alveolar macrophages. Paraffin sections of human lung tissue from healthy individuals (left column) and ex-smokers with COPD (right column) were immunostained with anti-OCTN2 antibody (brown) and counterstained with hematoxylin (blue). **a** and **b** Show expression of OCTN2 on the apical side of bronchial epithelium (arrow heads). **c** and **d** Show OCTN2 expression in bronchiolar epithelial cells (arrow heads). **e** and **f** Show positive staining of OCTN2 in alveolar macrophages (arrows) and in alveolar epithelial cells (arrow heads). **g** Confirms the OCTN2 expression in alveolar type II cells (TTF1 positive) (arrows) after co-staining with OCTN2 (green) and TTF1 (red) antibodies. Scale bar: 20μm

Expression of OCTN2 was similar to OCTN1 expression, and was found on the apical and lateral side (Fig. 5a and b) of the epithelial cells in the bronchial region. OCTN2 was also expressed in epithelial cells lining the bronchioles (Fig. 5c and d) in alveolar macrophages and type II cells (Fig. 5e-g).

**Fig. 6:**
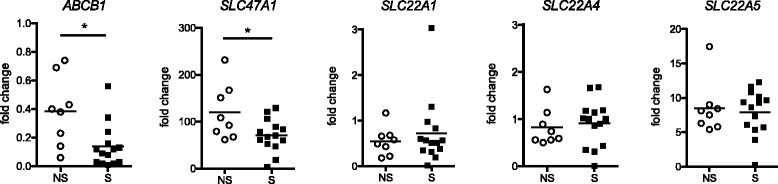
Differential expression of membrane transporters in BAL cells from healthy smokers and non-smokers. Normalized mRNA expression of selected genes, shown as fold change relative to the median expression in all subjects. Horizontal lines indicate median. NS: non-smokers, S. Smokers. Statistics calculated using Mann Whitney non-parametric test. * < 0.05

As all investigated membrane transporters were found to be expressed in alveolar macrophages in both COPD and healthy subjects (Table 2) we further analyzed their mRNA expression in isolated primary cells from bronchoalveolar lavage fluid (BAL cells), obtained from non-smokers and from smokers with normal lung function. *ABCB1* (P-gp), *SLC47A1* (MATE1), *SLC22A4* (OCTN1), and *SLC22A5* (OCTN2) were all expressed in BAL cells from both smokers and non-smokers (Fig. 6). As the isolated BAL cell samples contained mainly alveolar macrophages (median of 87.4 and 96.2% alveolar macrophages in non-smokers and smokers, respectively, see Table 1) the expression analyses thus confirm the expression of P-gp, MATE1, OCTN1 and OCTN2 in alveolar macrophages, as seen by IHC. We also analyzed the mRNA expression of *SLC22A1* coding for the organic cation transporter OCT1. Overall, *SLC47A1* (MATE1) showed the highest expression of the genes for transporters investigated, followed by *SLC22A5* (OCTN2)*, SLC22A4* (OCTN1)*, SLC22A1* (OCT1), and *ABCB1* (P-gp). Notably, the expression of *ABCB1* (P-gp) and *SLC47A1* (MATE1), was significantly downregulated in cells from smokers, compared to non-smokers, while *SLC22A1* (OCT1), *SLC22A4* (OCTN1), and *SLC22A5* (OCTN2) showed similar expression in the two groups (Fig. 6). There was a trend toward a negative correlation of the expression of *ABCB1* (P-gp) and *SLC47A1* (MATE1) with increased number of pack years and number of cigarettes smoked/day (Additional file 1: Figure S4). This further strengthens the evidence towards a relation between smoke exposure and reduced expression of these genes.

**Fig. 7:**
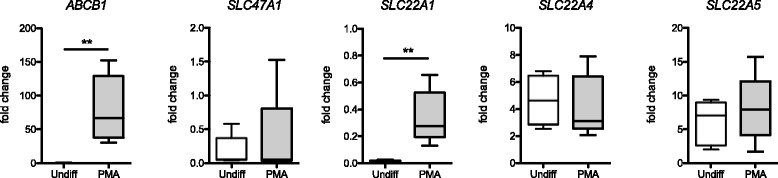
Differential expression of membrane transporters in the THP-1 cell line. Normalized mRNA expression of selected genes, after differentiation into macrophage-like cells, (PMA), or after incubation with media alone (undiff) for 48 h. Expression displayed as fold change, relative to the median expression in all samples. *N* = 5 samples/stimulation, performed in consecutive experiments. Horizontal lines indicate median, boxes indicate the interquartile range, and whiskers the minimum and maximum values, respectively. Statistics calculated using Mann Whitney non-parametric test. ** < 0.01

To further explore the direct role of cigarette smoke on the expression of membrane transporters in macrophages we used the human THP-1 cell line. THP-1 cells are cultured in a monocytic state and are differentiated into macrophage-like cells in the presence of PMA. Expression analysis revealed that the THP-1 cells express all the transporters analyzed (Fig. 7). Interestingly, we found that *ABCB1* (P-gp) and *SLC22A1* (OCT1) were almost exclusively expressed in THP-1 cells after PMA-induced differentiation to macrophages whereas these two transporters showed very low expression in the monocytic state (Fig. 7). In contrast, expression of *SLC47A1* (MATE1), *SLC22A4* (OCTN1) and *SLC22A5* (OCTN2) remained unchanged by the PMA treatment. In differentiated THP-1 cells, *ABCB1* (P-gp) showed the highest expression of the genes for transporters investigated, followed by *SLC22A5* (OCTN2), *SLC22A4* (OCTN1), *SLC22A1* (OCT1) and *SLC47A1* (MATE1). In order to investigate if inflammatory stimuli or cigarette smoke exposure influence the expression of membrane transporters, the differentiated cells were also stimulated with lipopolysaccharide (LPS), and cigarette smoke extract (CSE). These stimulations did however not induce any significant changes in expression, of the analyzed membrane transporters (Additional file 1: Figure S5).

**Fig. 8:**
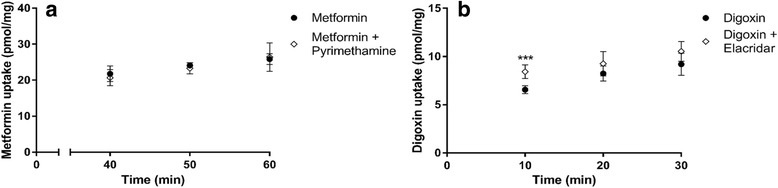
Investigation of the functional activity of MATE1 and P-gp in THP-1 cells using transporter inhibitors. Uptake in differentiated THP-1 cells 72 h after PMA induction overnight. **a** Metformin (11 μM) uptake with (◊) and without (●) pyrimethamine (1 μM) in the incubation and (**b**) digoxin (0.5 μM) uptake with (◊) and without (●) elacridar (10 μM) in the incubation. Results are given as mean ± S.D., *n* = 5 to 6 for each time point. *** *p* < 0.001

We further analyzed the functional expression of selected transporters in THP-1 cells differentiated by PMA, using known transporter substrates and inhibitors (Fig. 8). The organic cation transporter substrate metformin was taken up by differentiated THP-1 cells (Fig. 8a). In order to test the functional activity of MATE1 in THP-1 cells, pyrimethamine, a selective MATE1 inhibitor, was added to the cells before the addition of metformin. The uptake of metformin was however not significantly affected by the addition of pyrimethamine. This indicates that MATE-1 does not have a substantial functional expression in this assay, in line with the low mRNA expression of *SLC47A1* detected in these cells (Fig. 7).

Digoxin, a substrate for P-gp, was also taken up into differentiated THP-1 cells. Furthermore, the intracellular concentration of digoxin significantly increased in the presence of the P-gp inhibitor elacridar. The effect of elacridar indicates that the P-gp transporter is functionally active, secreting digoxin out of THP-1 cells in the absence of the inhibitor.

## Discussion

In the current study, we present for the first time the localization of MATE1 protein, an important drug transporter, in the human lung e.g. on the apical side of bronchial and bronchiolar epithelial cells, and in particular in alveolar macrophages and other immune cells. In addition, we demonstrate that P-gp, OCTN1 and OCTN2 proteins are expressed in epithelial and inflammatory cells in the lung including in alveolar macrophages, and confirmed mRNA expression of all these transporters, including MATE1 as well as OCT1, in BAL cells obtained from healthy subjects. Notably, mRNA encoding for P-gp (*ABCB1*) and MATE1 (*SLC47A1*) were down-regulated in BAL cells from smokers as compared to non-smokers whereas a difference was not observed in non-smoking COPD patients when compared to healthy volunteers. These findings are important to consider when developing drugs towards inflammatory lung disease. As a result of variability in transporter expression, drug disposition at the site of target, e.g. intracellularly in alveolar macrophages, could be distinct in various study populations, e.g. smokers compared to non-smokers or a healthy lung compared to an inflamed lung. Early phase clinical studies are often conducted in young healthy males and it is important to keep in mind when analyzing early biomarkers that drug disposition in this population might not completely reflect the situation in patients.

We recently showed that MATE1 mRNA (*SLC47A1*) was expressed in lung tissue from COPD patients and healthy subjects [7]. In the present study, we demonstrate MATE1 protein expression on the apical membrane of epithelial cells in bronchial and bronchiolar epithelium. Previously, MATE1 protein has mainly been studied in kidney and liver where it is located on the apical membrane of proximal tubule cells and hepatocytes, respectively [35].

In accordance with previous literature, OCTN1, OCTN2 and P-gp proteins were also expressed in epithelial cells in the bronchi [5, 6, 11, 21]. Several inhaled drugs such as β-adrenergic agonists and muscarinic antagonists are good substrates of organic cation transporters, and are dependent on these transporters to cross cell membranes, due to their polarity [6, 10]. It has been suggested that transport of β-adrenergic agonists and muscarinic antagonists by OCT1, OCTN1 and OCTN2, over the apical membrane of epithelial cells in the bronchi serves as the first step for these compounds to be transported across the epithelial cell barrier to their target receptors in the smooth muscle cells [11, 14]. However, the transporter that secretes these cationic compounds on the basolateral side of the airway epithelial cells has not yet been identified. In the renal proximal tubule cells, the basolateral expression of OCT2 works in concert with the apically expressed MATE1 to transport cationic drugs like cimetidine from blood to urine. Thus, MATE1 would be a plausible candidate to transport cationic inhaled drugs out of airway epithelial cells on the basolateral side. However, we here demonstrate that MATE1 is located on the apical side of bronchial and bronchiolar epithelial cells, thus making this role for MATE1 unlikely. Interestingly, in an in vitro model of bronchial epithelium using Calu-3 cells, it was recently demonstrated that ipratropium, a muscarinic antagonist that is a substrate for MATE1 [17], was not transported trans-vectorially through the cell layer but was instead both taken up and excreted on the apical side of the cells [36]. Although it is not known if MATE1 is expressed in Calu-3 cells, our finding of MATE1 on the apical side of the bronchial epithelium in the human lung indicates that is the case. Therefore, MATE1 may be responsible for the efflux of ipratropium on the apical side of Calu-3 cells. Thus, the mechanism of muscarinic antagonist and β-agonist absorption through the bronchial epithelial cell layer to the smooth muscle cells, in particular the efflux on the basolateral side, still remains to be clarified. Interestingly, expression of MATE1 protein, as well as the other investigated transporters OCTN1, OCTN2 and P-gp, was also found in immune cells, with particularly strong expression in alveolar macrophages. Both epithelial cells and macrophages form the surface that is exposed to a large variety of environmental factors, such as microorganisms, inhaled drugs, cigarette smoke and other pollutants, and function as a defense system. In addition, these cell types are key components of the pulmonary inflammatory response in COPD and other diseases. Expression of membrane transporters in immune cells, in the lung or other tissues, has not been as extensively studied. However, it is known that transporters are expressed in several types of immune cells, including T-cells, monocytes, macrophages, and dendritic cells [37]. Transporters are believed to be involved in active secretion of endogenous compounds in these cell types, for example inflammatory mediators such as prostaglandins, leukotrienes, sulfatides and cyclic nucleotides [37]. Additionally, cholesterol [38], cortisol, platelet activating factor and sphingosine 1-phosphate are substrates for P-gp, which is expressed in several immune cells [39–41] . Although not fully elucidated, there are thus several indications that membrane transporters play an active role in the lung immune response and in inflammatory diseases in the lung through their uptake and efflux of immunomodulating agents. Transporters may also be important for the disposal of drugs by inflammatory cells and may thus play a role in targeting cells involved in disease progression.

The mRNA expression of *ABCB1* (P-gp) and *SLC47A1* (MATE1) was significantly lower in BAL cells isolated from smokers as compared to never smokers. Notably, the decrease in P-gp and MATE1 expression correlated to the extent of smoking i.e. pack years or number of cigarettes/day. The regulation of drug transporters is often specific to each cell and tissue, leading to a differential expression pattern of transporters across tissues as well as a tissue specific response to endogenous and exogenous factors. Drugs and other xenobiotics have the capability to both induce and repress expression of transporter mRNA, by binding to nuclear hormone receptors [42], and P-gp is known to be upregulated via the nuclear receptors, pregnane X receptor (PXR) and constitutive androstane receptor (CAR) [42]. However, there is very limited knowledge about nuclear hormone receptor dependent regulation of MATE1 expression although hepatocyte nuclear factor 4α (Hnf4α) is likely essential for its expression in liver [43].

The reduced expression of *ABCB1* (P-gp) and *SLC47A1* (MATE1) in BAL cells from smokers as compared to non-smokers may be a result of different stimuli. Cigarette smoke contains a wide range of chemical substances known to directly interact with nuclear hormone receptors. This includes polyaromatic hydrocarbons, smoke components with the ability to activate the arylhydrocarbon receptor (AhR), resulting in strong induction of drug disposition genes e.g. *CYP1B1* in the lung [44]. Downregulation of MATE1, and other transporters, by cigarette smoke extract and the potent AhR ligand 2,3,7,8-tetrachlorodibenzo-p-dioxin (TCDD), has previously been seen in HepRG cells, a hepatocyte cell line [45]. However, regulation of P-gp by AhR has to our knowledge not been described in the literature, and no change in P-gp expression was observed in lung tissue from smokers or COPD patients when compared to healthy tissue [7, 46]. It is therefore probable that another mechanism regulates the observed downregulation of *ABCB1* (P-gp) expression, observed in this study. In addition, cytokines released during inflammation are known to down-regulate several CYP enzymes [47]. Interestingly, pro-inflammatory cytokines have been shown to downregulate MATE1 expression in synovial fibroblasts from human subjects with rheumatoid arthritis [48]. Similarly, P-gp was downregulated in an acute inflammation model using human peripheral blood mononuclear cells [49]. The mechanism of downregulation of P-gp and MATE1 mRNA that was observed in BAL cells from smokers needs to be further investigated. It is important to point out that expression regulation in disease is complex and may be affected by different factors, in the case of COPD by e.g. inflammatory mediators and smoke components.

The human monocyte cell line THP-1 is widely used as a model system for studying various mechanisms in monocytes and macrophages, and the cells adopt several key features of tissue macrophages after differentiation with PMA [50]. The PMA treatment induces the differentiation into proinflammatory macrophage-like cells that however retain the capability to be further stimulated by LPS [51] and other Toll Like Receptor ligands (data not shown). The THP-1 cell line has also been used for in vitro stimulations using cigarette smoke extract, and these studies have shown that smoke extract exposure induce a range of transcriptional changes, related to inflammation, oxidative stress and several other cellular processes [52], and can potentiate the LPS-induced NF-kB signaling [53]. The THP-1 cell line has also been used to study the expression and function of membrane transporters. Shimizu et al. studied *SLC22A4* (OCTN1) in the context of colitis, and described an increase in gene expression after PMA differentiation of THP-1 cells [54]. Perdomo and coworkers [55] detected P-gp protein in differentiated THP-1 cells, and found that benznidazole can further upregulate the protein levels of this protein. We analyzed the expression of several genes encoding for membrane transporter and found significant differences in *SLC22A1* (OCT1) and *ABCB1* (P-gp) mRNA levels, in differentiated versus undifferentiated cells. This indicates that the macrophage-like THP-1 cells, with several characteristics resembling tissue macrophages, may be more adapted to actively transport a variety of small compounds, compared to monocytes. We further stimulated the differentiated cells with LPS or CSE, though this did not significantly change the expression of the analyzed genes. While this model has some limitations, this suggests that short-term exposure to smoke or inflammatory stimuli is not sufficient to induce the downregulation of gene expression, that is seen for *ABCB1* (P-gp) and *SLC47A1* (MATE1) in the human primary BAL cells from smokers. Thus, additional signaling mechanisms, related to the long-term smoke exposure in the lungs of habitual smokers, are involved in this transcriptional regulation.

## Conclusions

We have demonstrated the expression of key drug transporters in pulmonary tissue and inflammatory cells that are targets for treatment of lung diseases such as COPD. We also observed a difference in transporter expression in BAL cells from smokers and non-smokers. Transporter proteins may affect drug disposition in lung and consequently the ability of drugs to reach their intracellular targets. Moreover, transporter proteins are involved in the uptake and release of endogenous mediators from inflammatory cells. Thus, understanding drug transporter expression and regulation is important in the development of successful new inhaled drug therapies.

## Additional file

Additional file 1: Supplemental data. (DOCX 6444 kb)

## Abbreviations

ABC: ATP-binding cassette
AW: Airway
BAL: Bronchoalveolar lavage
BALT: Bronchus-associated lymphoid tissue
CAR: Constitutive androstane receptor
COPD: Chronic Obstructive Pulmonary Disease
CSE: Cigarette Smoke Extract
DDI: Drug-drug interactions
FEV1: Forced expiratory volume in one second
FVC: Forced vital capacity
HEK: Human embryonic kidney
HKG: Housekeeping gene
Hnf4α: Hepatocyte nuclear factor 4α
IHC: Immunohistochemistry
LPS: Lipopolysaccharide
MATE1: Multidrug and toxin extrusion protein 1
MDR: Multidrug resistance protein
MRP: Multidrug resistance associated protein
OCT: Organic cation transporter
OCTN: Novel organic cation transporter
PBS: Phosphate buffered saline
P-gp: P-glycoprotein 1
PMA: Phorbol 12-myristate 13-acetate
PXR: Pregnane X receptor
qPCR: Quantitative real-time PCR
SLC: Solute Carrier
TCDD: 2,3,7,8-Tetrachlorodibenzo-p-dioxin
TTF1: Thyroid Transcription Factor1
