# Supplementary material for: Expression of MATE1, P-gp, OCTN1 and OCTN2, in epithelial and immune cells in the lung of COPD and healthy individuals
Source: Respir Res. 2018 Apr 20;19:68. doi: 10.1186/s12931-018-0760-9 (PMC5910606; doi:10.1186/s12931-018-0760-9)
Supplement: Supplementary file 1 — Supplemental data. (DOCX 6444 kb) [file 12931_2018_760_MOESM1_ESM.docx]

**Supplemental data**

**Expression of MATE1, P-gp, OCTN1 and OCTN2, in epithelial and immune cells in the lung of COPD and healthy individuals**

Tove Berg, Tove Hegelund-Myrbäck, Johan Öckinger, Xiao-Hong Zhou, Marie Brännström, Michael Hagemann-Jensen, Viktoria Werkström, Janeric Seidegård, Johan Grunewald, Magnus Nord, Lena Gustavsson

**Cigarette Smoke Extract (CSE).** CSE was produced in-house according to a standardized protocol. In short, a 2R4F research cigarette from Kentucky Tobacco Research & Development Center, University of Kentucky, was fixed to a silicone tube attached to a vacuum flask with RPMI cell culture medium, and connected to a mechanical pump. A 3-way valve fitted between the pump and the vacuum flask was used to manually regulate the inflow of smoke from the lit cigarette (i.e. “puffs” on the cigarette). The cigarette was smoked in short puffs (2 seconds) followed by a rest for 8 seconds, and combustion of the cigarette down to the filter required 10-12 puffs/cigarette. The mainstream smoke was collected in 7.5 mL RPMI/cigarette (4 cigarettes to 30 ml RPMI). After collection of the cigarette smoke into the media, pH was adjusted to 7.4 and the solution was filtered through a 0.2 µm filter, forming the 100% CSE containing 3.57 µg/ml of nicotine. Nicotine concentration in CSE was measured at ABS Laboratories, Hertfordshire, UK. The CSE was stored in aliquots at -80°C until use.

**THP-1 cell culture for uptake experiments.** THP-1 cells, grown in suspension, were maintained in RPMI 1640 medium (Sigma-Aldrich, St. Louis, MO, USA), supplemented with 10% FCS, 2 mM L-Glutamine, 1 mM sodium pyruvate and 100 U/mL Penicillin and Streptomycin. Except for the RPMI medium, all cell culture reagents were purchased from Invitrogen Life Technologies (Paisley, UK). For uptake experiments, 500 000 cells/well were seeded in 24-well plates (Falcon, Corning, Tewksbury, MA, USA) and differentiated with 10 ng/ml PMA (Sigma-Aldrich, St. Louis, MO, USA) to adherent macrophages overnight. Experiments were conducted 48 or 72 hours post PMA-removal.

**THP-1 cell culture for expression analysis.** Human monocytic cell line THP-1 were grown in suspension at 37°C in 5% CO2 in RPMI-1640 supplemented with HEPES (25 mL), L-glutamine (2 mM), sodium pyruvate (1 mM), heat inactivated fetal calf serum (5%) penicillin (100U/ml), streptomycin (100μg/ml) and β-mercaptoethanol (4.35 μg/ml). All reagents from Sigma-Aldrich (St. Louis, USA). THP-1 cells were differentiated to macrophage-like cells using 10 ng/ml PMA (Phorbol 12-myristate 13-acetate; Sigma-Aldrich) for 48h, cell concentration 0.5x106 cells/ml in tissue culture plates. After differentiation cells were gently washed with sterile PBS (37°C), and replenished with fresh culture medium. Cigarette smoke extract (CSE), and/or LPS (10 ng/ml, E. coli K12, Invivogen, Toulouse, France) was added to the culture as indicated and cells were stimulated for additional 12h. Thereafter supernatants were removed and cells lysed and homogenized in RLT Plus buffer (Qiagen, GmbH, Hilden, Germany) according to the manufacturer’s instructions, and lysates stored at -80°C, until RNA extraction and expression analysis as described.

**Table S1** Description of subjects in clinical study where human tissue material was obtained

|  | Age (years) | Sex | Pack years | FEV1 (L) | FEV1/FVC (%) |
| --- | --- | --- | --- | --- | --- |
| COPD Patients | |  |  |  |  |
| 1 | 64 | F | 60 | 0.50 | 38 |
| 2 | 62 | F | 50 | 0.52 | 26 |
| 3 | 61 | M | 40 | 0.77 | 39 |
| 4 | 61 | M | 50 | 0.60 | 24 |
| 5 | 62 | M | 50 | 0.90 | 32 |
| 6 | 65 | F | 30 | 0.49 | 32 |
| 7 | 63 | M | 45 | 1.00 | 37 |
| Healthy Controls | |  |  |  |  |
| 1 | 46-65 | F | Non-smoker |  |  |
| 2 | 46-65 | F | Non-smoker |  |  |
| 3 | 46-65 | M | Non-smoker |  |  |

**FEV1:** [Forced expiratory volume in one second](http://copd.about.com/od/glossaryofcopdterms/g/FEV1.htm), **FVC:** Forced vital capacity**, Packyears:** number of *cigarettes* smoked per day × number of years smoked)/20

**Supplementary Figures.**


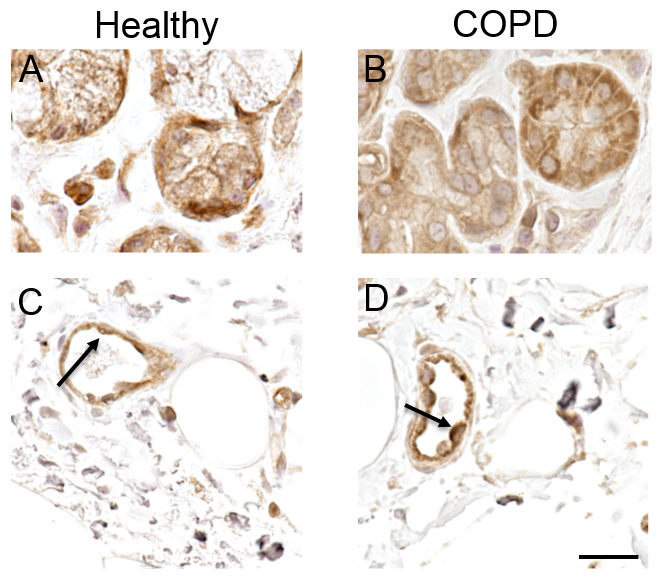


**Fig S1**. Paraffin sections of human lung tissue from healthy individuals (left column) and ex-smokers with COPD (right column) were immunostained with anti- Pgp antibody (brown) and counterstained with hematoxylin (blue). A, B) Pgp positive signals in seromucous glands. C and D) Pgp expression in endothelial cells(arrows). Scale bar: 20μm


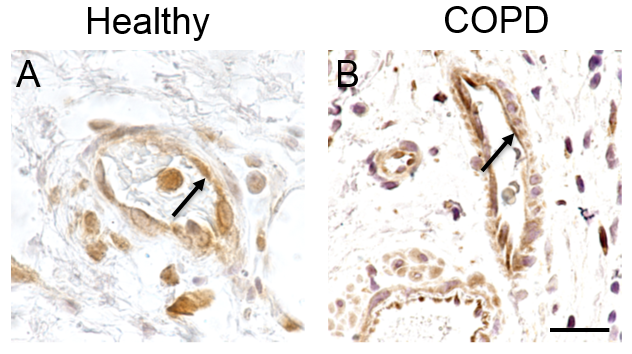


**Fig S2**. Paraffin sections of human lung tissue from healthy individuals (left column) and ex-smokers with COPD (right column) were immunostained with anti- MATE1 antibody (brown) and counterstained with hematoxylin (blue). A, B) MATE1 positive signals in endothelial cells(arrows). Scale bar: 20μm


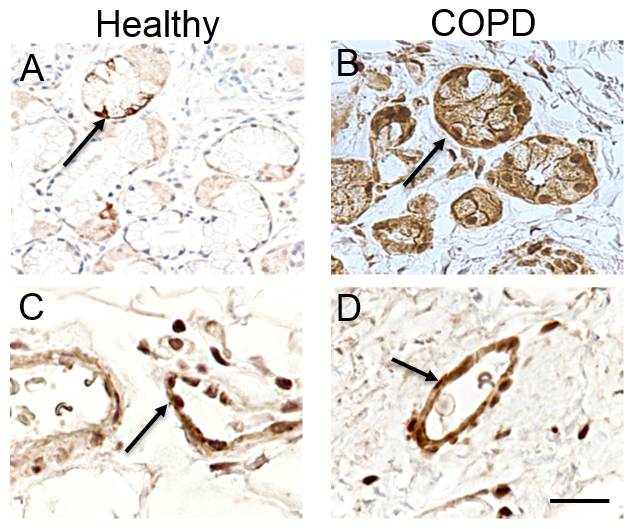


**Fig S3**. Paraffin sections of human lung tissue from healthy individuals (left column) and ex-smokers with COPD (right column) were immunostained with anti- OCTN1 antibody (brown) and counterstained with hematoxylin (blue). A, B) OCTN1 positive signals in submucosa glands (arrows). C and D), OCTN1 expression in endothelial cells(arrows). Scale bar: 20μm


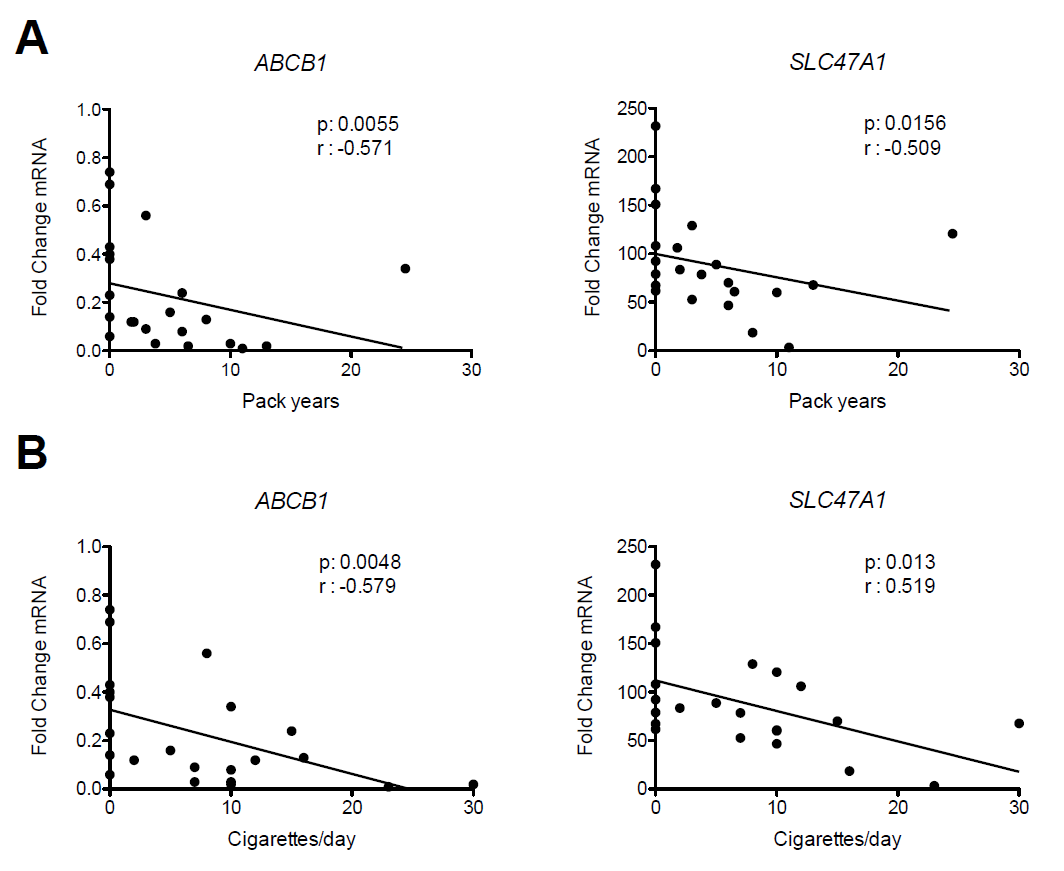


**Fig S4.** Expression of membrane transporters is correlated with smoking habits in individuals with normal lung function. Normalized mRNA expression of selected genes was correlated to (A) pack years or (B) cigarettes smoked per day, at time of bronchoscopy. Correlation calculated using Spearman’s rank correlation.


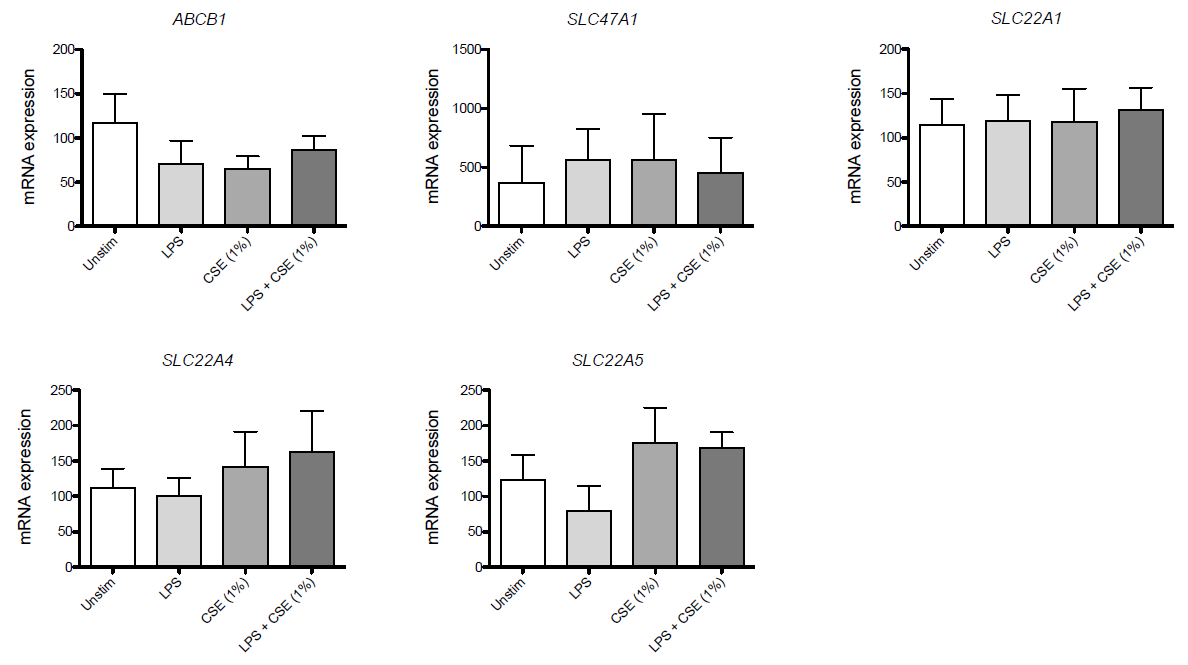


**Fig S5.** Expression of membrane transporters is not altered by *in vitro* stimulation. Differentiated THP-1 cells were incubated for 12 hours with medium alone (unstim), LPS (10ng/ml), 1% cigarette smoke extract (CSE) in cell culture media, or both LPS and CSE. Normalized mRNA expression shown relative to unstimulated cells. Bars show the mean and error bars indicate the SEM. N=4-5 samples/ stimulation


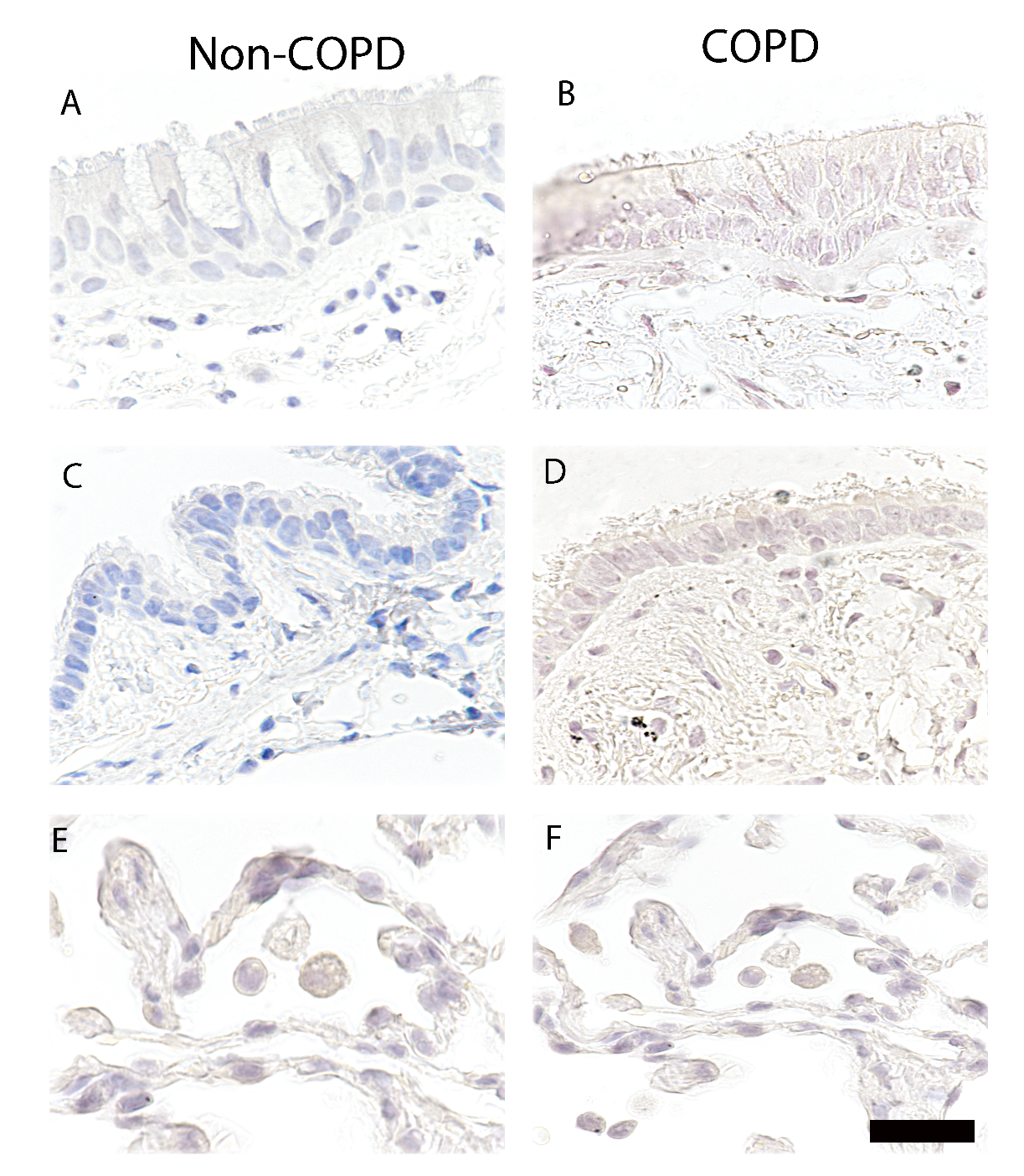


**Fig S6**: Rabbit isotype IgG control for the antibodies against P-gp and MATE1. Bronchi from control donor (A) and COPD patient (B), bronchioles from control donor (C) and COPD patient(D). Parenchyma from control donor (E) and COPD patient (F). Scale bar: 20μm


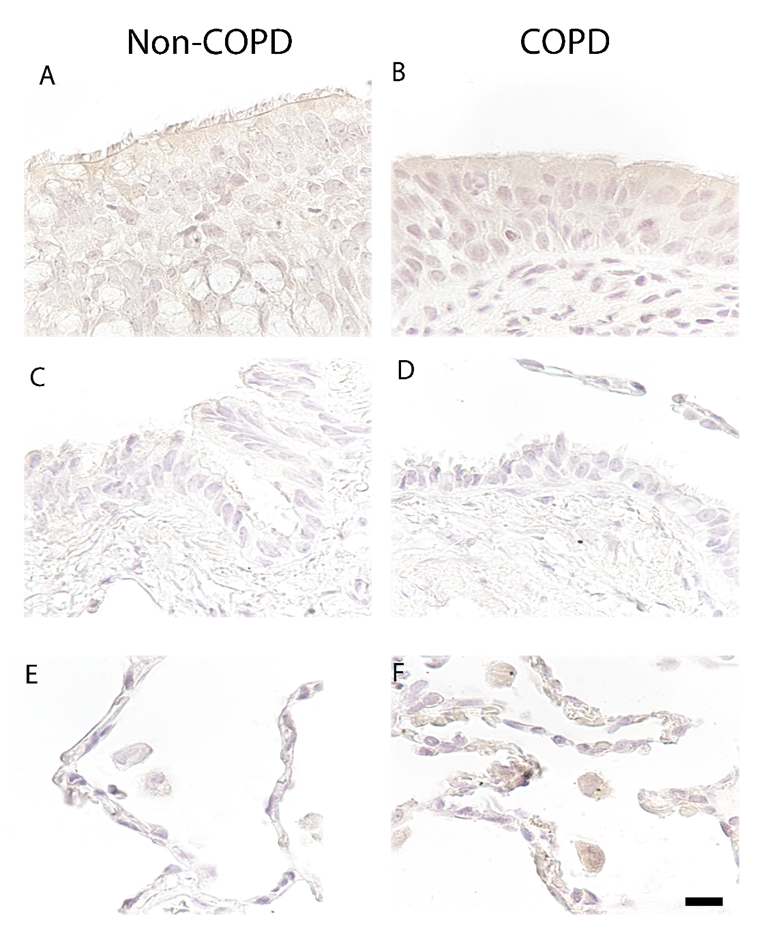


**Fig S7:** Goat isotype IgG control for the antibody against OCTN1 and OCTN2. Bronchi from control donor (A) and COPD patient (B), bronchioles from control donor (C) and COPD patient(D). Parenchyma from control donor (E) and COPD patient (F). Scale bar: 20μm


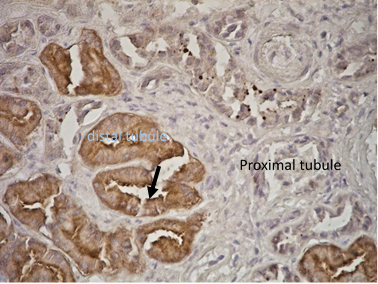


**Fig S8:** Reference tissue (kidney) for OCTN1 antibody. Validation of the goat anti OCTN1 polyclonal antibody (sc-19819) used in our experiments, in formalin fixed and paraffin embedded human kidney tissue section (purchased from Medical Solutions). It showed a positive signal in the distal tubules and significant staining in the apical side of the epithelial cells (arrow). The signal is absent from the proximal tubules, in agreement with literature.


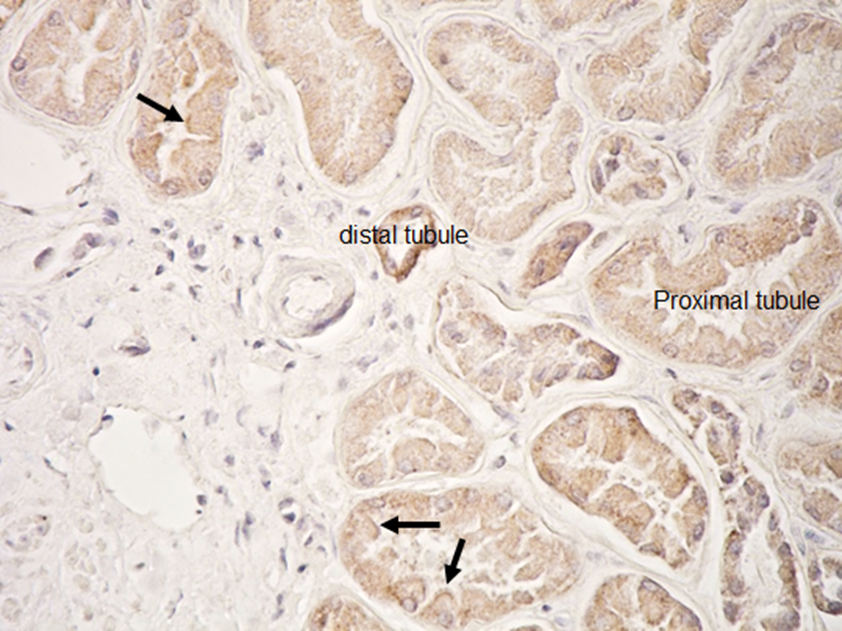


**Fig S9:** Reference tissue (kidney) for the OCTN2 antibody. Validation of the goat anti OCTN2 polyclonal antibody (sc-19822) used in our experiments, in formalin fixed and paraffin embedded human kidney tissue section (purchased from Medical Solutions). It showed a positive signal in the tubular epithelial cells and significant staining in the apical side of the proximal tubular epithelium (arrows). The is signal is absent from surrounding interstitial cells and matrix structures, in agreement with the literature.
